# High-density lipoproteins during sepsis: from bench to bedside

**DOI:** 10.1186/s13054-020-02860-3

**Published:** 2020-04-07

**Authors:** Sébastien Tanaka, David Couret, Alexy Tran-Dinh, Jacques Duranteau, Philippe Montravers, Anna Schwendeman, Olivier Meilhac

**Affiliations:** 1 Université de La Réunion, INSERM, UMR 1188 Diabète athérothombose Réunion Océan Indien (DéTROI), Saint-Denis de La Réunion, France; 2 AP-HP, Service d’Anesthésie-Réanimation, CHU Bichat-Claude Bernard, Paris, France; 3 CHU de La Réunion, Saint-Pierre de la Réunion, France; 4 Inserm UMR1148, Laboratory for Vascular Translational Science Bichat Hospital, Paris, France; 5 AP-HP, Service d’Anesthésie-Réanimation, Hôpitaux Universitaires Paris-Sud, Université Paris-Sud, Hôpital de Bicêtre, Le Kremlin-Bicêtre, France; 6 Laboratoire d’étude de la Microcirculation, “Bio-CANVAS: biomarkers in CardioNeuroVascular DISEASES” UMRS 942, Paris, France; 7 Inserm UMR1152. Physiopathologie et Epidémiologie des Maladies Respiratoires, Paris, France; 8 Department of Pharmaceutical Sciences, College of Pharmacy, University of Michigan, Ann Arbor, MI 48109 USA; 9 Biointerfaces Institute, University of Michigan, Ann Arbor, MI 48109 USA

**Keywords:** Endothelium, High-density lipoprotein (HDL), Inflammation, Intensive care unit (ICU), Lipopolysaccharide (LPS), Sepsis, Sepsis therapy

## Abstract

High-density lipoproteins (HDLs) represent a family of particle characterized by the presence of apolipoprotein A-I (apoA-I) and by their ability to transport cholesterol from peripheral tissues back to the liver conferring them a cardioprotective function. HDLs also display pleiotropic properties including antioxidant, anti-apoptotic, anti-thrombotic, anti-inflammatory, or anti-infectious functions. Clinical data demonstrate that HDL cholesterol levels decrease rapidly during sepsis and that these low levels are correlated with morbi-mortality. Experimental studies emphasized notable structural and functional modifications of HDL particles in inflammatory states, including sepsis. Finally, HDL infusion in animal models of sepsis improved survival and provided a global endothelial protective effect. These clinical and experimental studies reinforce the potential of HDL therapy in human sepsis. In this review, we will detail the different effects of HDLs that may be relevant under inflammatory conditions and the lipoprotein changes during sepsis and we will discuss the potentiality of HDL therapy in sepsis.

## Background

Despite a better comprehension of this entity, sepsis remains a pathology with a high rate of morbi- and mortality worldwide [1]. Pathophysiological pathways involved in sepsis are complex, including pro- and anti-inflammatory signaling along with major non-immunological responses such as cardiovascular, neuronal, autonomic, hormonal, and metabolic responses, as well as activation of coagulation [2]. Recent definitions and consensus underline that sepsis is clearly defined as a life-threatening organ dysfunction caused by deregulated host response to infection [1]. The failure of therapies using specific anti-inflammatory treatments may be due to the complexity of sepsis signaling, modulation and pattern, and finally to the poor understanding of the pathophysiology.

High-density lipoproteins (HDLs) represent a family of particles characterized by their ability to transport cholesterol from peripheral tissues back to the liver that confers to them an anti-atherogenic protective effect. Many experimental studies emphasize on other pleiotropic properties of HDLs, including anti-inflammatory, anti-apoptotic, or antioxidant functions [3, 4]. Furthermore, HDLs have the property to bind and neutralize lipopolysaccharide (LPS) [5] that could be particularly relevant in septic conditions. Other studies have also demonstrated that infusion of reconstituted HDL (rHDL) or HDL mimetic particles decreased morbi- and mortality in animal models of sepsis [6].

Because of their pleiotropic protective effects, HDLs may represent a potential future therapeutic target to be explored in human sepsis. The objectives of this present review are to describe HDL properties that can play a role in sepsis and to summarize clinical and experimental studies involving HDL during sepsis.

## HDL structure and diversity

Lipoproteins are macromolecular particles composed by proteins including apolipoproteins associated with a phospholipid layer containing a lipid core consisting in free cholesterol, cholesterol esters, and triglycerides [7]. They are classified according to their density, which is proportional to their protein content: chylomicrons, very low-density lipoproteins (VLDLs), intermediate-density lipoproteins (IDLs), low-density lipoproteins (LDLs), and high-density lipoprotein (HDLs), being the particles with the highest protein content.

Specifically, HDLs are defined by a density ranging from 1.063 to 1.21. To characterize this lipoprotein population, different techniques are used and underline the heterogeneity of HDL subclasses. Ultracentrifugation allows isolation of HDL fractions into HDL2a, HDL2b, and HDL3, whereas electrophoresis on gradient gels separates HDL particles by size (HDL2a, HDL2b, HDL3a, HDL3b, HDL3c). Two-dimensional gel electrophoresis has been used to separate HDL populations according both to their charge and size into small pre-β and large α1-α4 HDL particles. Rosenson et al. have suggested a new classification defining 5 HDL subclasses on the basis of physical and chemical properties and named very large, large, medium, small, and very small HDL particles [8]. Finally, HDL particles may be classified according to their major lipoprotein contents [7].

As compared to other lipoproteins, HDLs are also characterized by their abundant protein content and protein diversity. Although HDL particles are mainly composed by ApoA-I, proteomic analysis has emphasized the numerous proteins that are transported by HDLs, including enzymes, acute phase response proteins, complement system proteins, and proteinase inhibitors [9].

## HDL metabolism and main function: reverse transport of cholesterol (Fig. 1)

One major function of HDL metabolism is the reverse cholesterol transport (RCT) permitting the efflux of cholesterol from peripheral cells back to the liver that confers to HDL a cardiovascular protective effect [10, 11].

**Fig. 1:**
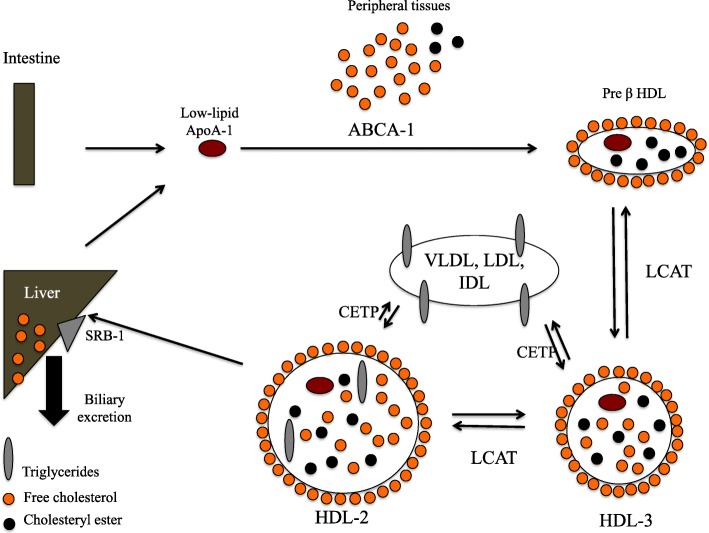
Reverse transport of cholesterol

The first step of the biosynthesis of HDL particles is the release of lipid-free or lipid-poor apolipoprotein A-I by the liver and intestine. Lipid-poor apoA-I will then acquire phospholipids and free cholesterol via their interaction with ATP-binding cassette transporter A1 (ABCA1), which is a membrane transporter protein mainly expressed by macrophages and hepatocytes, but also in the brain and other tissues. With the acquisition of these lipids, HDL particles become discoidal, forming disk-shaped pre-β [12]. Then, lecithin-cholesterol acyltransferase (LCAT), which is activated by apoA-1, will esterify free cholesterol. Cholesterol esterification leads to a structural modification of HDL particles, which become smaller, dense, and spherical (HDL_3_). HDL_3_ particles continue to accept free cholesterol and apolipoproteins forming bigger and lipid-rich particles named HDL_2_. HDL_2_ and HDL_3_ have the possibility to exchange esterified cholesterol against triglycerides (TG) from other lipoproteins (VLDL, IDL, LDL). TG molecule is bigger than esterified cholesterol, which participates in increasing the size of HDL particles. This important remodeling system is performed by cholesteryl ester transfer protein (CETP).

HDL_2_ is then removed from the circulation by the liver via the scavenger receptor class B type I (SRB1), leading to the degradation of cholesterol esters by hepatocytes and their excretion into the bile. This interaction with SRB1 releases lipid-poor Apo A-I, which initiates a new cycle of RCT. The second mechanism of the degradation of cholesterol esters is through CETP which can transfer cholesterol esters from HDL particles to TG-rich lipoproteins (VLDL and LDL) and then cholesterol finally reaches the liver when LDL particles are taken up by the liver via the LDL receptor.

## HDL cholesterol (HDL-C) levels vs HDL RCT functionality

The current paradigm that HDLs are protective for cardiovascular diseases relies mainly on the results of the Framingham Study that reported a 2–3% decrease in coronary artery disease risk with each increase by 1 mg/dL in HDL-C [13]. Most of the epidemiological studies have evaluated the cholesterol concentration in the non-precipitable lipoprotein fraction of plasma/serum, assumed to be “HDL-C”; however, this concentration only poorly reflects the capacity of HDL particles to reverse transport the cholesterol from peripheral tissues back to the liver. Cholesterol efflux capacity can be evaluated in vitro, using macrophages, and has been shown to be inversely correlated to carotid intima-media thickness and CAD risk, independently of the HDL-C levels [14]. This functional RCT assay is also inversely related to the incidence of cardiovascular events in a population-based cohort [15]. A wealth of genetic and interventional studies suggests that increasing HDL-C levels is not sufficient to limit CVD risk. On the one hand, Mendelian randomization studies report that single nucleotide polymorphism modulating HDL-C levels such as variants of endothelial lipase [16] or phospholipid transfer protein [17] did not impact on CVD risk prediction (see meta-analysis in [18]). On the other hand, clinical trials using cetrapibs (CETP inhibitors) showed that, albeit raising HDL-C levels, these molecules were unable to improve cardiovascular outcomes [19], suggesting that evaluation of HDL functionality should be an important readout for testing new therapies.

## Pleiotropic effects of HDL (Fig. 2)

### Lipopolysaccharide (LPS) and lipoteichoic acid (LTA) binding and neutralization properties of HDL (Fig. 3)

LPS is the major component of the outer membrane of Gram-negative bacteria. In noncapsulated strains, LPS is exposed on the cell surface. Numerous studies have demonstrated that all lipoproteins (chylomicrons, VLDL, LDL, and HDL) are capable to bind Gram-negative LPS. However, it is clearly established that LPS preferentially binds HDL particles relative to other lipoproteins [20]. Levels et al. have incubated different labeled LPS chemotypes with delipidated or normal plasma and determined LPS fluorescence profiles by high-performance gel permeation chromatography [21]. These authors demonstrated that LPS binding to lipoproteins is highly specific and that HDLs have the highest binding capacity for LPS as compared to that of other lipoproteins. Furthermore, Levine et al. have shown that transgenic mice expressing human Apo A-I displayed lower cytokine levels after LPS injection compared to control mice [22]. This interaction is facilitated by the action of specific lipid transfer proteins such as CETP, PLTP, and LPS-binding protein (LBP) [23]. These specific proteins permit the transfer of LPS to lipoproteins. For example, as described by Vesy et al., mainly LBP but also PLTP can extract LPS from bacterial membranes and transfer it to HDL particles in human serum [24].

**Fig. 2:**
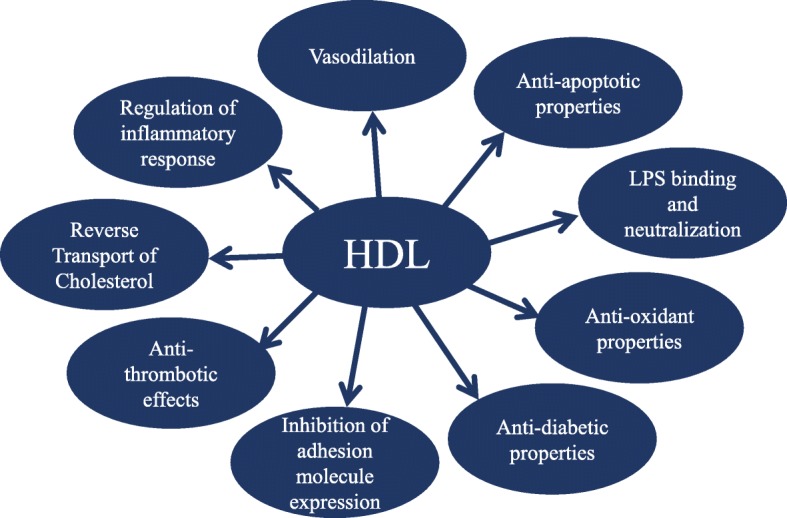
Pleiotropic properties of HDL

**Fig. 3:**
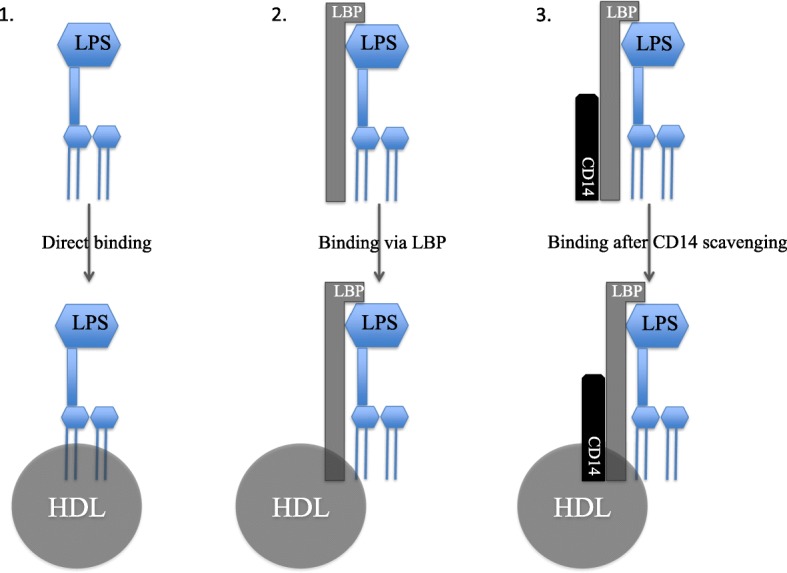
Binding and neutralization of LPS by HDL: 3 potential pathways

However, the mechanisms underlying the association between LPS and HDLs remain unclear: LPS lipid A diglucosamine-phosphate region seems to be the key part of LPS molecules allowing the interaction between LPS and HDL particles [25]. Furthermore, HDL particles are probably not sufficient to neutralize the biologic activity of LPS [5]. Theoretically, LBP is required to form a complex between LPS and CD14 able to bind HDL particles, permitting its neutralization.

Gram-positive do not have LPS, but lipoteichoic acid (LTA), an amphiphilic molecule formed by a hydrophilic polyphosphate polymer linked to a neutral glycolipid which is a major immunostimulatory component for these bacteria. The association of LTA with lipoproteins shows a striking similarity with that of LPS. HDL seems to have the highest affinity for LTA [26]. Grunfeld et al. have demonstrated that HDL can inhibit macrophage activation by LTA [27].

### Inhibition of adhesion molecule expression

During sepsis and inflammatory states, the adhesion of leukocytes to the endothelium is a key step allowing their migration within the tissues [2]. Cockerill et al. have shown that physiological concentrations of human isolated HDLs inhibited in vitro the expression of leukocyte adhesion molecule expression (V-CAM-1, ICAM-1, E-selectin) on endothelial cells induced by pro-inflammatory cytokines [28]. Reconstituted HDLs also inhibited the TNFα-induced expression of V-CAM-1. The same authors have confirmed this action in vivo in a porcine model of inflammation [29]. Another study emphasized the fact that rHDLs attenuated adhesion molecule expression in a rodent model of endotoxin shock whereas pretreatment of LPS-injected rats with rHDL limited the expression of P-selectin and ICAM-1 caused by endotoxin in the kidney [30]. Another possible mechanism of HDL protective effects is the modulation of transcription factors, which may decrease adhesion molecule expression via the inhibition of nuclear factor NF-kB activity [31].

### Regulation of inflammatory response in macrophages

HDLs may also be a key modulator of inflammatory response in macrophages [32, 33]. One important finding is that HDLs stimulate the transcriptional regulator ATF3, which downregulates inflammatory pathways that may in turn decrease the inflammatory response in case of sepsis [34]. Moreover, Zhu et al. have shown in murine macrophages that human serum amyloid A (SAA) dramatically upregulates the expression and secretion of a group of phospholipases (sPLA2-IIE and sPLA2-V), which are late pro-inflammatory mediators family [35]. In this in vitro study using purified HDL, HDL dose-dependently attenuated SAA-induced secretion of both sPLA2-IIE and sPLA2-V. Lastly, Suzuki et al. demonstrated that HDLs inhibit a subset of LPS-stimulated macrophage genes that regulate the type I interferon response, independently of sterol metabolism, raising the possibility that regulation of macrophage transcriptome by HDLs might link innate immunity to cardioprotection [36].

### Microvascular vasodilation and endothelium repair

HDLs have the property to stimulate the endothelial NO synthase (eNOS), which is involved in inhibiting the adhesion of monocytes to the endothelium and promotes microvascular vasodilation [3]. This upregulation of eNOS expression is dependent on SR-B1 receptor. Specific subclasses of HDL particles bind with different affinity to SR-B1. For example, lipid-poor and lipid-free apoA-I have a lower affinity for this receptor, leading a reduced vasodilation property [37]. HDL particles can also stimulate the production of prostacyclin, which is a powerful vasodilator. Kontush et al. have shown that HDL_3_ sub-fraction had a better capacity to improve NO production than HDL_2_ because of its enrichment of sphingosine-1-phosphate (S1P), which stimulates the production of prostacyclin [38]. Moreover, in a population of coronary artery disease patients, Sattler et al. described that reduced S1P content in HDL particles abrogated their vasodilatory capacity, contributing to HDL dysfunction [39]. S1P-loading in vitro and in vivo increased eNOS activation and improved the vasodilatory property of HDLs [39, 40].

### Anti-thrombotic effects

HDLs stimulate the endothelial production of NO and prostacyclin which are inhibitors of platelet activation. Furthermore, HDLs prevent platelet hyper-reactivity by limiting intraplatelet cholesterol overload and the interaction of apoA-I on platelet ABCA1 and SRB1 receptors. This leads to the inhibition of the pro-coagulation cascade and subsequent clot formation [41]. HDLs may also prevent the endothelial thrombotic activation, by promoting prostacyclin and Cox2 production and by reducing the expression of tissue factor and of adhesion molecules [42]. A direct anti-thrombotic property of HDLs has been reported, via the reduction of thrombus formation in a study involving a mutant form of human ApoA-I [43]. Furthermore, HDLs can enhance particular anticoagulant activity (inactivation of active factor V) exerted by activated protein C and protein S [44]. A direct antiplatelet effect was also described in vivo and in vitro by reducing platelet aggregation induced by collagen, ADP, or thrombin [45].

### Antioxidant properties

Paraoxonase (PON1) is an HDL-associated esterase, protecting lipoproteins against oxidation. PON1 is able to hydrolyze lipid peroxides and especially oxidized cholesteryl esters and phospholipids [46]. Moreover, PON1 also hydrolyzes phosphatidylcholines into lysophosphatidylcholines, which improve the bactericidal activity of neutrophils potentially resulting in a protective effect in experimental sepsis [47]. Shih et al. have demonstrated that PON1-deficient mice are susceptible to oxidative stress and that HDLs isolated from these mice were unable to prevent LDL oxidation [48]. The global antioxidant effect of HDLs is evaluated via their capacity to inhibit LDL oxidation. This property consists in the transfer of oxidized lipids from oxidized LDL (oxLDL) (hydroperoxides and lysophosphatidylcholine) to HDL particles and by the inactivation of oxidized lipids. Lastly, HDLs are able to limit oxidation by decreasing ROS production via the inactivation of neutrophil NADPH oxidase [49]. Oxidation is an important phenomenon observed in sepsis [50], and reduced HDL antioxidant function may participate in sepsis progression/severity.

### Anti-apoptotic properties

HDLs exert a protective effect on endothelial cell apoptosis by interfering with both receptor-mediated death signaling and mitochondrial apoptotic pathways. Suc et al. have demonstrated that HDLs have the capacity to inhibit apoptosis of endothelial cells induced by oxLDL [51]. In oxLDL-induced apoptosis, HDLs were shown to interact with the endoplasmic reticulum. As underlined by Nofer et al., Akt signaling, a major anti-apoptotic pathway, is stimulated by HDLs in a model of endothelial cell apoptosis (HUVECs) [52]. In this study, HDLs were also capable of inhibiting caspase-3 and caspase-9 activation. Theilmeier et al. have shown in vitro and an in vivo mouse model of myocardial ischemia/reperfusion that HDLs and its sphingolipid component S1P reduced cardiomyocyte apoptosis [53]. Moreover, a direct infusion of native HDLs or a S1P receptor agonist reduced cardiomyocyte apoptosis, myocardial injury, and the size of myocardial infarction [54, 55]. Taken together, these anti-apoptotic effects and more globally endothelium protective effects of HDLs may limit the progression and the severity of sepsis, in which endothelium aggression play a pivotal role [3].

### Anti-diabetic properties

Diabetic patients usually have a dyslipoproteinemia characterized by increased triglycerides and low HDL-C levels, with TG-enriched HDL resulting from a CETP-mediated interchange of TG from TG-rich lipoproteins to HDLs [56]. HDL particles have been reported to display anti-diabetic properties by improving β cell insulin secretion through ABCA1 and ABCG1: Brunham et al. have shown that mice with specific inactivation of ABCA1 in β cells had markedly impaired glucose tolerance and defective insulin secretion but normal insulin sensitivity [57]. This was also demonstrated in carriers of disruptive mutations in ABCA1 Tangier disease patients [58]. Numerous experimental studies have also shown that HDLs improve insulin sensitivity [59–61]; Han et al. have demonstrated that ApoA-I possesses protective effects against diabetes via activation of AMP-activated protein kinase and that ApoA-I deletion in mice led to increased fat mass and impaired glucose tolerance [60]. In humans, HDLs from diabetic patients are dysfunctional [62]. An acute infusion of reconstituted HDs in thirteen patients with type 2 diabetes mellitus reduced plasma glucose by increasing plasma insulin levels and activating AMP-activated protein kinase in skeletal muscle [63].

## Lipoprotein changes during human sepsis

In critically ill patients and especially in septic patients, a reduction in lipid and lipoprotein levels is well documented [64]. Clinical observations have shown that circulating levels of HDLs decrease during the acute phase of inflammatory state and especially during a sepsis [65–68]. van Leeuwen et al. have demonstrated that lipoprotein levels rapidly drop up to 50% of initial concentrations in patients with severe sepsis and that this rapid reduction was particularly marked in LDL and HDL cholesterol levels [65]. We have compared HDL profiles between septic and trauma patients [69]. Although inflammation is exacerbated in these two entities, HDL-C levels were lower in septic patients, whereas their concentration was not altered in case of trauma. Interestingly, in a study involving healthy subjects, low HDL levels were correlated with increased inflammatory response to endotoxin challenge compared with subjects with normal or high HDL levels [70].

During sepsis, several hypotheses may provide explanation for this dramatic reduction, including an acute over-consumption of HDL particles, a decrease in liver HDL synthesis, especially in case of hepatic failure, and/or an increased clearance following an upregulation of SRB1 [71]. In the context of sepsis characterized by a severe inflammation-induced capillary leakage, HDL particles may easily be redistributed from the intravascular to the extravascular compartment [65]. Another hypothesis would be a decrease of ApoA-I due to its replacement by serum amyloid A (SAA) in HDL particles at the early phase of sepsis [72]. SAA is able to displace ApoA-I from the surface of HDL particles, generating free ApoA-I, which is cleared faster by the kidney, thus contributing to reducing HDL-C levels and functionality [73].

The majority of these observational studies emphasized the negative correlation between HDL concentration and mortality [66, 67]. A low HDL concentration at day 1 was significantly associated with an increased mortality and adverse clinical outcomes with a cut-off ranging from 20 to 25 mg/dL [67]. In a prospective study including 151 consecutive septic patients, a low apoA-I concentration was independently related to 30-day mortality [66]. Interestingly, a recent study has shown that variations in genes involved in HDL metabolism could contribute to changes in HDL-C levels but also to clinical outcomes following a sepsis [74]. These authors have identified a rare missense variant in CETP (rs1800777-A) that was associated with significant reductions in HDL-C levels during sepsis. In this study, carriers of the A allele had an increased mortality, more organ failure, and greater need for organ support compared to non-carriers.

However, other studies failed to find any link between HDL levels and mortality. We did not find any correlation between mortality and HDL concentration but a poor outcome defined as death or SOFA score > 6 at day 3 was associated with lower HDL levels in a population of 50 septic patients [69]. van Leeuwen et al. did not find any correlation between lipoprotein concentrations in survivors and non-survivors [65]. Whereas triglycerides were associated with mortality in septic patients, no correlation was found with other lipoproteins and especially with HDL concentration [75].

HDL levels were also associated with morbidity in several studies [75, 76]: for example, in a 2-year follow-up of patients with septic shock, low HDL levels were associated with increased risk of sepsis-associated acute kidney injury (AKI) and a decrease in estimated glomerular filtration rate (eGRF) [76].

Moreover, because sepsis still remains an important cause of mortality and morbidity, early biomarkers could be useful to establish a diagnosis of sepsis and also to indicate its severity. To date, there is no biomarker that fulfills these objectives in terms of sensibility and specificity. Some authors have suggested that HDL levels could represent an early marker of sepsis severity. Chien et al. underlined the power of HDL and Apo A-I levels at day 1 to predict the overall 30-day mortality rate [67]. At ICU admission, according to Barlage et al. study [66], low HDL-C (AUC of the ROC 0.6, *p* = 0.049) and low apoA-1 (AUC of the ROC 0.604, *p* = 0.041) levels were predictive of sepsis-related mortality. In a cohort of 200 patients enrolled at the emergency department with clinically suspected sepsis, comparing to different variables such as white blood cell count, lactate, or platelets, HDL concentration was the best predictor of both development of multiorgan dysfunction syndrome and 28-day mortality [68].

## HDL function in inflammatory state

Acute inflammation alters both lipoprotein composition and metabolism resulting in reduced anti-inflammatory properties, in particular for HDL particles [72]. Numerous remodeling in HDL composition are currently described during these inflammatory states, such as apoA-I dissociation from the particles, reduction of esterified cholesterol, and decrease in HDL-associated enzymes (LCAT, CETP, or PON1 for example) [65]. It is well established that the presence of blood endotoxins modulates HDL particle composition. For example, during infection and inflammation, SAA displaces apoA-I within HDL particles, thus becoming the predominant apolipoprotein of septic HDLs [72]. This observation was also described in inflammatory states following cardiac surgery with cardiopulmonary bypass [77].

HDL-associated PON-1 is also altered in septic condition. Novak et al. have described a dramatic decrease in PON-1 activity in septic patients versus controls [78]. The oxidative environment induced by sepsis could result in an increased binding of free radicals to PON-1, leading to an overall decreased plasma activity of this enzyme. Platelet-activating factor-acetylhydrolase (PAF-AH) activity, which is able to inhibit the formation of non-esterified fatty acid hydroperoxides from oxLDL, was also reported to be altered in septic condition [79]. With the reduction in plasma PAF-AH and PON1 activities during sepsis, HDLs display blunted protective effects against LDL oxidation [80].

Proteomic studies underlined the diversity of HDL-associated proteins in normal conditions [9]. HDL proteome is clearly modified during inflammatory conditions [81] and in particular in sepsis state [82]. Sharma et al. evaluated the host proteome response in septic patients secondary to community-acquired pneumonia (CAP) [82]. This analysis has shown alteration in the cytoskeleton, cellular assembly, movement, lipid metabolism, and immune response in septic patients. Focusing on apolipoproteins, the authors demonstrated a decrease of some apolipoproteins (ApoA-I, ApoA-II, ApoA-IV, ApoB, ApoC-I, ApoC-II, ApoC-III, and ApoE). A recent study on the lipidome showed a decrease in phospholipid concentration in hospital-acquired pneumonia [83].

Reverse cholesterol transport is also impaired in inflammatory conditions. Specifically, de la Llera et al. have shown that in twenty healthy adults, an endotoxemia induced by LPS (3 ng/kg) administrated as an intravenous bolus infusion led to a reduced capacity of HDLs to efflux cholesterol in vitro [72]. Interestingly, when comparing the 10 oldest septic patients to the ten oldest healthy subjects, the cholesterol efflux was significantly reduced in septic patients (24 ± 1.2%) compared to control subjects (31.5 ± 1.0%) [84].

In this context, dysfunctional HDLs has emerged as a new concept in sepsis as it was well documented in other diseases such as atherosclerosis, stroke, or auto-immune pathology [77–79, 81, 85]. It is now clearly established that HDLs become dysfunctional during sepsis and are associated with a poor outcome.

HDL size is also affected during inflammation and infection [72]. de la Llera et al. have shown that LPS infusion in healthy volunteers led to a decrease of small- and medium-size particles without any change in the total number of HDL particles [72]. We have shown that HDL levels are dramatically decreased in the acute phase of septic shock and that there is a shift toward large HDL particles [86]. Another study underlined that both HDL size and HDL-C concentration were independently associated with coronary artery disease risk [87]. In a recent study involving 402 patients who underwent carotid MRI assessment for lipid-rich necrotic core plaques, HDL particle size was significantly associated with HDL efflux capacity suggesting that differences in HDL efflux capacity may be due to structural differences in HDL particles [88]. However, because different techniques are used to assess HDL size, correlation of size with function should be exploited with caution.

In the light of these observations, HDL-C concentration is probably not sufficient to characterize HDL function. Size modification and HDL remodeling leading to changes in composition appear to be more relevant to make HDL dysfunction more helpful in clinical practice. In this context, since both quantitative and qualitative modifications of HDL particles are observed in septic conditions, supplementation with functional HDL in experimental pre-clinical models should be tested.

## HDL-based therapies in experimental sepsis studies

Because of HDL pleotropic properties, the drastic reduction of HDL concentration and the dysfunction observed during inflammatory states, reconstituted HDLs or apoA-I mimetic peptide infusion may represent a potential therapy in sepsis [6, 22, 30, 71, 89–93]. Moreover, ApoA-I knockout mice exhibited reduced LPS neutralization in serum relative to controls, supporting the concept of rising functional HDL levels as a therapeutic approach for sepsis [94].

Several apoA-I mimetic peptides such as peptide 4F (Ac-DWFKAFYDKVAEKFKEAF-NH2) have been synthetized and are able to bind phospholipids and associate with native HDL particle [95]. Zhang et al. have shown in a rat model of sepsis that infusion of apoA-I decreased plasma IL-6 concentration, improved the cardiac function, and reduced the mortality [96]. Other studies have demonstrated that this peptide attenuated kidney, heart, vascular, and lung injury and improved survival in experimental models of sepsis [90, 97].

Reconstituted HDLs (rHDLs) were also tested in septic models [30, 91]. Different types of rHDLs have been experimented, but rHDLs are usually prepared with a 1/150 ratio of apoA-1 to soybean phosphatidylcholine (PC) [98]. For example, McDonald et al. have shown that rHDL infusion limited renal injury and dysfunction and reduced the degree of histological tissue injury in the lung, liver, and intestine [30]. Recently, in 3 different experimental mouse models of sepsis, we demonstrated that infusion of rHDL improved survival, reduced inflammation in both plasma and organs, and decreased bacterial count [99]. Interestingly, immunohistological analysis of septic lungs emphasized that apoA-I reached and accumulated in pulmonary tissue in rHDL-injected mice, suggesting that HDL particles can locally exert their protective effects. Moreover, ^111^Indium bacterial labeling provided a potential hepatic bacterial clearance possibly promoted by HDL uptake.

An additional table underlines these different experimental sepsis studies in more detail (see Additional file 1).

## rHDLs or mimetic peptide infusion in human sepsis?

In the cardiovascular field, rHDLs have been tested in atherosclerotic and diabetic patients. Thirteen patients with type 2 diabetes mellitus received rHDLs and saline in a randomized crossover design study [100]. Four and 72 h after rHDL infusion, the anti-inflammatory properties of isolated HDL increased. Interestingly, there was also an enhancement of cholesterol efflux.

In case of coronary atherosclerotic disease, Tardif et al. have investigated the effects of rHDLs (CSL111®) on the plaque burden as assessed by intravascular ultrasound (IVUS) [101]. In this randomized controlled trial, 183 patients were randomized to receive CSL111® (40 mg/kg or 80 mg/kg) or placebo. Concerning the primary outcome of the study, there was no significant reduction in percentage change in atheroma volume or nominal change in plaque volume in CSL111® group compared with placebo.

In pre-clinical studies, the ability of HDLs to bind endotoxin has been well correlated with their phospholipid content [22]. These results motivated the development of a protein-free phospholipid emulsion containing phosphatidylcholine, soybean oil, and sodium cholate in order to bind and eliminate LPS. A double-blind placebo-controlled study has enrolled 20 volunteers receiving *Escherichia coli* endotoxin infusion of either an emulsion of 92.5% of phosphatidylcholine and 7.5% of triglyceride or placebo [102]. A lower neutrophil count and TNFα and IL-6 levels were measured in patients who received the emulsion.

This encouraging result involving volunteers led to perform a randomized placebo-controlled phase II multicenter trial evaluating a phospholipid emulsion infusion in Gram-negative severe sepsis. However, the Lipid Infusion and Patient Outcomes in Sepsis (LIPOS) study failed to reduce 28-day all-cause mortality or the onset of new organ failure [103]. Timing of administration, no standardized protocol of care in this international study recruiting in 31 countries, and the heterogeneity of the recruited patients may explain these disappointing results.

To date, no randomized study using rHDLs or mimetic peptides in septic patients has been carried out. However, Pajkrt et al. have tested the effects of rHDLs in human endotoxemia [104]: 8 healthy male volunteers were enrolled in a double-blind crossover randomized placebo-controlled study. rHDLs given as a 4-h infusion at the dose of 40 mg/kg dramatically reduced the endotoxin-induced inflammatory response: rHDL infusion reduced the endotoxin-induced clinical symptoms (less chills, myalgia, backache, or vomiting) and importantly reduced the release of TNFα, IL-6, and IL-8 cytokines. Moreover, rHDL infusion was associated with a downregulation of CD14, the main LPS receptor on monocytes. The same team also reported that rHDL infusion can affect the fibrinolytic activity and can directly influence platelet function by reducing platelet aggregation leading a modification of the procoagulant state associated with endotoxemia [105].

## Conclusion

To summarize, in addition to its well-documented role in reverse transport of cholesterol, HDLs display numerous pleiotropic effects such as LPS neutralization, endothelial protection, and antioxidant and anti-apoptotic properties. Inflammation states and especially sepsis decrease dramatically HDL levels and alter their composition, metabolism, and function. These findings strongly support the therapeutic potential of rHDL or HDL mimetic peptide infusion in sepsis. With an improvement of survival, experimental studies involving rHDLs or HDL mimetic peptides are encouraging. However, further experimental studies are needed to better characterize this new concept of HDL dysfunction that is markedly associated with a poor outcome. A better comprehension of the function of these particles should encourage the medical and scientific community to initiate clinical trials aiming at testing the effect of a HDL therapy in human sepsis.

## Supplementary information

**Additional file 1.** HDL-based therapies in experimental sepsis studies. The table presents different experimental studies testing reconstituted HDLs or mimetic peptide by notifying the type of animal and model used, the type of product and dose, the modes and the timing of administration.

## Data Availability

Not applicable.

## Abbreviations

ABCA1: ATP-binding cassette A1
ABCG1: ATP-binding cassette sub-family G member 1
AKI: Acute kidney injury
ApoA-I: Apolipoprotein A-I
CAP: Community-acquired pneumonia
CETP: Cholesteryl ester transfer protein
eGRF: Estimated glomerular filtration
eNOS: Endothelial NO synthase
HDL: High-density lipoprotein
HDL-C: HDL cholesterol
IDL: Intermediate-density lipoprotein
IVUS: Intravascular ultrasound
LBP: LPS-binding protein
LCAT: Lecithin-cholesterol acyltransferase
LDL: Low-density lipoprotein
LPS: Lipopolysaccharide
LTA: Lipoteichoic acid
oxLDL: Oxidized LDL
PAF-AH: Platelet-activating factor acetylhydrolase
PC: Phosphatidylcholine
PLTP: Phospholipid transfer protein
PON-1: Paraoxonase-1
RCT: Reverse cholesterol transport
SAA: Serum amyloid A
SOFA: Sepsis-related organ failure assessment
SRB1: Scavenger receptor class B type 1
VLDL: Very low-density lipoprotein
