# Supplementary material for: High-density lipoproteins during sepsis: from bench to bedside
Source: Crit Care. 2020 Apr 7;24:134. doi: 10.1186/s13054-020-02860-3 (PMC7140566; doi:10.1186/s13054-020-02860-3)
Supplement: Supplementary file 1 — Additional file 1. HDL-based therapies in experimental sepsis studies. The table presents different experimental studies testing reconstituted HDLs or mimetic peptide by notifying the type of animal and model used, the type of product and dose, the modes and the timing of administration. [file 13054_2020_2860_MOESM1_ESM.pdf]

**Table S1: HDL-based therapies in experimental sepsis studies**

| Study | Animal | Sepsis | Administration route if LPS | LPS dosing | rHDL/peptide | rHDL/peptide administration route | rHDL/peptide dosing | Timing |
|---|---|---|---|---|---|---|---|---|
| **Datta. J Clin Exp Cardiolog 2011** (1) | Rat | LPS (*E. coli* 026:B6) | IP | 10mg/kg | 4F peptide | IP | 10mg/kg | LPS + 4F simultaneously |
| **Kwon. J Trauma 2012** (2) | Rat | LPS (*E. coli* 026:B6) | IV | 10mg/kg | 4F peptide | IP | 10mg/kg | 10' after LPS injection |
| **Sharifov. Plos One 2013** (3) | Rat | LPS (*E. coli* 026:B6) | IP | 30mg/kg | 4F peptide | IV | 10mg/kg | 1h after LPS injection |
| **Dai. J lipid Res 2010** (4) | Rat | LPS (*E. coli* 0111:B4) | IV | 10mg/kg | 4F peptide | IP | 10mg/kg | LPS + 4F simultaneously |
| **Levine. PNAS 1993** (5) | Mice | LPS (*E. coli* 026:B6) | IP | 10mg/kg | - 18A peptide<br>- rHDL | IV | - 80mg/kg<br>- 80mg/kg | 15' before sepsis Pretreatment |
| **Casas. Am Surg 1996** (6) | Rabbit | *E. coli* | IV | $4.10^9$ CFU/kg | rHDL (red cross Bern) | IV | 75mg/kg | Before sepsis Pretreatment |
| **Casas. J Surg Res 1995** (7) | Rabbit | Blended Gram neg LPS | IV | Not stated | rHDL (red cross Bern) | IV | - 25mg/kg<br>- 75mg/kg | Before sepsis Pretreatment |
| **McDonald. Shock 2003** (8) | Rat | LPS (*E. coli* 0127:B8) | IV | 6mg/kg | rHDL (red cross Bern) | IV | Not stated | 5' before LPS Pretreatment |
| **Zhang. Biol Chem 2015** (9) | Rat | LPS | IV | Not stated | ApoA-I Milano | IV | 40mg/kg | Before sepsis Pretreatment |
| **Zhang. AJPHCP 2009** (10) | Rat | CLP | | | 4F peptide | IP | 10mg/kg | 6h after CLP |
| **Guo. J Biol Chem 2013** (11) | ApoA-I KO + tg mice | CLP | | | none | none | none | none |
| **Moreira. AJPRICP 2014** (12) | Rat | CLP | | | 4F peptide | IP | 10mg/kg | 6h after CLP |
| **Tanaka. Anesthesiology 2020** (13) | Mice | - CLP<br>- *E. coli* IAI76 intraperitoneal injection<br>- *P. aeruginosa* pneumonia | | | rHDL (CSL Behring) | IV | 40mg/kg | 2h after sepsis |

1. Datta G, Gupta H, Zhang Z, Mayakonda P, Anantharamaiah GM, White CR. HDL Mimetic Peptide Administration Improves Left Ventricular Filling and Cardiac output in Lipopolysaccharide-Treated Rats. J Clin Exp Cardiolog. 22 déc 2011;2(172).

2. Kwon WY, Suh GJ, Kim KS, Kwak YH, Kim K. 4F, apolipoprotein AI mimetic peptide, attenuates acute lung injury and improves survival in endotoxemic rats. J Trauma Acute Care Surg. juin 2012;72(6):1576‑83.

3. Sharifov OF, Xu X, Gaggar A, Grizzle WE, Mishra VK, Honavar J, et al. Anti-inflammatory mechanisms of apolipoprotein A-I mimetic peptide in acute respiratory distress syndrome secondary to sepsis. PLoS ONE. 2013;8(5):e64486.

4. Dai L, Datta G, Zhang Z, Gupta H, Patel R, Honavar J, et al. The apolipoprotein A-I mimetic peptide 4F prevents defects in vascular function in endotoxemic rats. J Lipid Res. sept 2010;51(9):2695‑705.

5. Levine DM, Parker TS, Donnelly TM, Walsh A, Rubin AL. In vivo protection against endotoxin by plasma high density lipoprotein. Proc Natl Acad Sci USA. 15 déc 1993;90(24):12040‑4.

6. Casas AT, Hubsch AP, Doran JE. Effects of reconstituted high-density lipoprotein in persistent gram-negative bacteremia. Am Surg. mai 1996;62(5):350‑5.

7. Casas AT, Hubsch AP, Rogers BC, Doran JE. Reconstituted high-density lipoprotein reduces LPS-stimulated TNF alpha. J Surg Res. nov 1995;59(5):544‑52.

8. McDonald MC, Dhadly P, Cockerill GW, Cuzzocrea S, Mota-Filipe H, Hinds CJ, Miller NE, Thiemermann C: Reconstituted high-density lipoprotein attenuates organ injury and adhesion molecule expression in a rodent model of endotoxic shock. Shock 2003, 20(6):551-557.

9. Zhang X, Wang L, Chen B. Recombinant HDL (Milano) protects endotoxin-challenged rats from multiple organ injury and dysfunction. Biol Chem. janv 2015;396(1):53‑60.

10. Zhang Z, Datta G, Zhang Y, Miller AP, Mochon P, Chen Y-F, et al. Apolipoprotein A-I mimetic peptide treatment inhibits inflammatory responses and improves survival in septic rats. Am J Physiol Heart Circ Physiol. août 2009;297(2):H866-873.

11. Guo L, Ai J, Zheng Z, Howatt DA, Daugherty A, Huang B, et al. High density lipoprotein protects against polymicrobe-induced sepsis in mice. J Biol Chem. 21 juin 2013;288(25):17947‑53.

12. Moreira RS, Irigoyen M, Sanches TR, Volpini RA, Camara NOS, Malheiros DM, et al. Apolipoprotein A-I mimetic peptide 4F attenuates kidney injury, heart injury, and endothelial dysfunction in sepsis. Am J Physiol Regul Integr Comp Physiol. 1 sept 2014;307(5):R514-524.

13. Tanaka S, Genève C, Zappella N, Yong-Sang J, Planesse C, Louedec L, et al. Reconstituted High-density Lipoprotein Therapy Improves Survival in Mouse Models of Sepsis. Anesthesiology. 2020 Feb 20.
